# Diversity of Cellulolytic Microorganisms Associated with the Subterranean Termite *Reticulitermes grassei*

**DOI:** 10.3390/jof9030294

**Published:** 2023-02-24

**Authors:** Juan Carbonero-Pacheco, José Aguilar, María Carmen Raya, Antonio Trapero, Miquel Gaju-Ricart, Carlos Agustí-Brisach

**Affiliations:** 1 Department of Agricultural Chemistry, Edaphology and Microbiology, Agrifood Campus of International Excellence CeiA3, Edif. C6, 14071 Córdoba, Spain; 2 Department of Agronomy (DAUCO, Unit of Excellence María de Maeztu 2020-23), Campus de Rabanales, Edif. C4, 14071 Córdoba, Spain; 3 Department of Zoology, Facultad de Ciencias, Universidad de Córdoba (UCO), Campus de Rabanales, Edif. C1, 14071 Córdoba, Spain

**Keywords:** cellulolytic, enzymatic index, fungi, identification, subterranean termites

## Abstract

*Reticulitermes grassei* is a subterranean termite species that forages on woody structures of the Iberian Peninsula, and is often a building and crops pest. A total of 23 microorganisms associated with the activity of *R. grassei* were isolated from colonized ecosystems in southern Spain. They were morphologically and molecularly characterized, with fungi being the most prevalent ones. The fungi showed high values of optimum growth temperature, suggesting that they could be able to survive and develop in warm regions. Their cellulolytic activity was tested in carboxymethylcellulose (CMC) agar, concluding that all fungal isolates produce cellulases, and the enzymatic index (EI) was revealed in CMC agar with Gram’s iodine solution, with *Penicillium citrinum* showing the highest EI and *Trichoderma longibrachiatum* the highest mycelial growth rate on CMC. A preliminary microorganism dispersion assay was carried out with the termites, concluding that these insects may have a positive influence on fungal dispersion and the subsequent colonization of new substrates. Our study suggests that fungi associated with *R. grassei* may potentially be of interest in biotechnological fields such as biofuel production and the food industry.

## 1. Introduction

Lignocellulose, the most abundant biomass on earth, is the most predominant component of the wood in terrestrial ecosystems. Due to the fact that plants produce around 180 billion tons of cellulose annually, cellulose is considered an inexhaustible source of raw material for many products [1], and it is the largest reservoir of organic carbon on Earth [2]. In nature, the degradation of cellulosic biomass is performed by mixtures of hydrolytic enzymes collectively known as cellulases. The cellulases include endo-acting (endoglucanases) and exo-acting (cellobiohydrolases) enzymes, which act in a synergistic manner in biomass-degrading microbes [3]. A broad range of microorganisms including fungi and bacteria can degrade cellulose and other plant cell wall components, with fungi being the main cellulase producer [4,5]. 

Several organisms have developed these enzymes to break down the cellulose of living or decaying plant material [3,6], as well as in the plants themselves. They have a potential value in biofuel production from plant debris [7]. Currently, a considerable diversity of species and environments are being screened for cellulolytic enzymes that possess improved characteristics, a process for which the reliable detection and quantification of cellulase activity is essential. 

Cellulose breakdown is a feature of many aerobic, facultatively anaerobic, and obligately anaerobic bacteria and fungi [8,9]. It is well known that the metabolism, physiology, and functional enzyme systems of these cellulolytic microorganisms are responsible for the largest flow of carbon in the biosphere [3,6,10], with important industrial and environmental implications [4,5].

For most animals, lignocellulose is a nutritionally poor food source that is highly resistant to enteric degradation. However, termites can digest lignocellulose with high efficiency [11]. These insects play important roles in lignocellulose bio-recycling in terrestrial tropical and subtropical ecosystems [12]. Indeed, they are considered a major decomposer in the detritus food chain. This ability relies on their own cellulases [13] combined with the microbes present in their gut. So-called lower termites, the subterranean termites belonging to Rhinotermitidae, i.e., *Reticulitermes grassei* (Isoptera), do not build mounds, but nest in or around partially buried wood and forage over large areas through an extensive gallery system [12,14]. This could contribute to the fact that lower termites are associated with a larger diversity of microorganisms than higher termites (Termitidae). Lower termites’ gut symbiotic microbiota can include microorganisms from the three domains Archaea, Bacteria, and Eucarya. The bacterial groups from the intestinal systems are mainly affiliated to the proteobacteria, a major phylum of Gram-negative bacteria, the Gram-positive groups Firmicutes and Actinobacteria, the Bacteroides/Flavobacterium branch, and the spirochetes. The Archaea are represented by methanogens. The eukaryotic groups consist of protozoa, yeasts, and fungi. Intestinal bacteria are involved in the degradation of cellulose, hemicellulose, and aromatic compounds, as well as nitrogen fixation [15]. Finally, higher termites apparently degrade cellulose using only their own enzymes, because of their lack of symbiotic protists [16]. 

Therefore, there is a large diversity of fungi associated with termites. Their presence associated with these insects has often been classified as beneficial because these symbiotic associations help in the carbon processing and nitrogen fixation from the breakdown of lignocellulosic components [17]. A broad diversity of microorganisms has already been isolated from termites, including genera of fungi such as *Acremonium*, *Alternaria*, *Aspergillus*, *Fusarium*, *Mucor*, *Neurospora*, *Penicillium*, *Rhizopus,* and *Trichoderma* from *Reticulitermes* spp. [17,18] and some cellulolytic bacteria such as *Acinetobacter* sp., *Bacillus cereus*, *Chryseobacterium kwangyangense,* and *Enterobacter aerogenes*, from the gut of *Coptotermes curvignathus* [19]. Recent studies show the broad diversity of filamentous fungi and yeasts associated with the activity of termites, and discuss the role of these fungal symbionts as lignocellulose decomposers that improve termite nutrition [20], while their tunnelling activity facilitates the spread of these microorganisms [21]. Thus, the objectives of this work were to isolate and characterize the microorganisms associated with the subterranean termite *R. grassei* in southern Spain, and to determine and quantify their cellulolytic activity. Additionally, the dispersion of microorganisms by termites was also preliminarily evaluated in the current study.

## 2. Materials and Methods

### 2.1. Field Sampling, Insect Collection, and Microorganism Isolation

#### 2.1.1. Field Sampling

Three ecosystems colonized by the subterranean termite *R. grassei* were sampled across the forest areas around the city of Cordoba (Andalusia region, southern Spain; UTM coordinates “30S 379133E 4710295N; 30S 348732E 4197825N; 30S 348394E 4197822N”) between spring and autumn 2018.

The sampled termites were found near a broad diversity of forest trees including *Eucalyptus*, *Pinus,* and *Populus* spp. Two types of samples were collected: (i) Degraded wood: wood from trees showing typical symptoms of degradation caused by *R. grassei* was collected, placed in plastic bags, and kept at 4 °C until processing in the laboratory; and (ii) Termites: baiting stations with corrugated cardboard [22] were placed in each of the three sampled fields described above to capture subterranean termites. The cardboard was previously sterilized at 100 °C for 24 h to avoid non-termite-related microorganisms of degradation caused by *R. grassei.*

#### 2.1.2. Microorganism Isolation

Once in the laboratory, microorganism isolation was conducted from wood samples as well as directly from the collected termites. From wood samples, small wood fragments from the degraded area were surface-disinfected by dipping them into a commercial bleach (Cl at 50 g L^–1^) solution at 10% (*v*/*v*) in sterile water for 2 min, after which they were air-dried on sterile filter paper for 15 min, and plated onto nutrient agar (NA, peptone 0.5%; yeast extract 0.3%; NaCl 0.5%; agar 2% and deionized water, autoclaved for 21 min at 121 °C) and agar sabouraud (AS; Difco^®^ Laboratories, Detroit, MI, USA) to make the isolation of both bacterial and fungal microorganisms easier. From termites, samples of 10 specimens were immersed in 1 mL of phosphate-buffered saline (PBS solution; NaCl, 8 g; KCl, 0.2 g; Na_2_HPO_4_, 1.44 g; KH_2_PO_4_, 0.24 g, in 800 mL of distilled H_2_O. pH adjusted to 7.4 with HCl, and then distilled H_2_O added to 1 L) for 1 min. Subsequently, aliquots of 100 µL of the PBS suspension were plated onto NA and AS. All Petri dishes were incubated at 27 ± 0.5 °C in the dark until colonies were large enough to be examined. The growing colonies were isolated on NA or AS to obtain axenic cultures.

### 2.2. Microorganism Dispersion by the Termites under Controlled Conditions

To evaluate the role of *R. grassei* in the dispersion of microorganisms and their interaction, two complementary assays were conducted. 

*Assay I.* Microorganism dispersion by *R. grassei*: groups of 150 termite workers of at least the third stage [23], collected from the bait stations, were placed into plastic containers (17.0 × 10.5 × 3.0 cm) in contact with two pieces (2.0 × 2.0 × 1.0 cm^3^) of sapwood of *Pinus pinea* or *Populus alba,* or *filter paper* [Whatman^®^ 1 Qualitative (Cat No 1001 110) of 110 mm diameter] as a food source. They were previously sterilized by means of kiln-drying at 60 °C for 48 h. This procedure is commonly used to study wood preferences in termites [24,25,26,27] because this temperature preserves the wood’s properties [28]. To maintain humidity, sterilized vermiculite (100 °C in oven for 24 h) was added into the plastic containers and then moistened with sterile distilled water in 1:3 proportion (*w*/*v*) following the methods of Gallardo et al. [29], and five replicates were conducted.

Plastic containers were randomly distributed in a room-temperature chamber at 26 ± 1 °C and 80 ± 5% relative humidity (RH) in the dark for one week. After that, degraded cellulose sources by *R. grassei* were collected and incubated for one week in the same conditions described above, but without the presence of termites. This procedure was carried out to observe the growth and development of the microorganisms dispersed by the termites on the food sources. Observed microorganisms in *Assay I* were isolated on NA or AS plates.

*Assay II.* Interaction between *R. grassei* and microorganisms: a complementary assay to assess the interactions between *R. grassei* and the microorganisms dispersed by them in the laboratory assays was conducted. The same cellulose sources (wood or filter paper) previously exposed to termites in *Assay I*, but now covered with microorganisms, were exposed again to a new group of 150 termites in the same conditions described for *Assay I*. The evolution of the fungal structures developed on the food sources by the activity of *R. grassei* was periodically observed under a stereoscopic microscope at 24, 48, and 120 h of termite exposure. Five replicates were conducted.

### 2.3. Microorganism Isolates

Among all the microorganisms isolated from the different sampled material, bacteria could be differentiated from fungi as they appeared shiny or filamentous, respectively. According to their appearance in culture, the isolates were firstly grouped as fungi (17 isolates) or bacteria (6 isolates). To obtain pure cultures, the fungal and bacterial colonies were transferred to AS and to NA, respectively. All the studied isolates are registered and maintained at 4 °C in the dark in the collection of the Department of Microbiology at the University of Cordoba (UCO), Spain.

### 2.4. Molecular Characterization

#### 2.4.1. DNA Extraction

Genomic DNA was obtained from the 17 fungal isolates and six bacterial isolates included in this study. All of them were actively grown on potato dextrose agar (PDA, Difco^®^ Laboratories, Le Pont de Claix, France) at 27 ± 0.5 °C in the dark for 7 days. Mycelial tissues and bacterial cells were ground using a FastPrep^®^-24 grinder machine (MP Biomedicals, Santa Ana, CA, USA). Subsequently, DNA extractions were performed using an E.Z.N.A.^®^ Fungal DNA Kit (OMEGA BioTek, Norcross, GA, USA) following the manufacturer’s instructions. A MaestroNano^®^ spectrophotometer (MaestroGen, Hsinchu City, Taiwan) was used to determine the concentration and purity of the extracted DNA.

#### 2.4.2. PCR Analysis

For the identification of fungal isolates, the ITS region (ITS1-5.8S rDNA-ITS2) [30] was amplified. Additionally, part of the beta-tubulin (TUB) [31] gene and the partial 28S rDNA gene (LSU) [32,33] were amplified to confirm the identity of some fungal isolates with ambiguous identification for the ITS marker. The identification of bacterial isolates was conducted by amplifying the 16S rDNA gene [34] In all cases, the PCRs were performed in a total volume of 25 µL containing the following mixture: 20 ng of genomic DNA, 5 µL of 5× My Taq Reaction Buffer, 0.4 μM each primer and 0.13 µL of My Taq DNA Polymerase (Bioline). A negative control was included in all PCRs using ultrapure water (PCR quality) instead of DNA. The primer pairs and PCR cycling programs used to amplify each gene are shown in Table 1. All PCRs were carried out in a MyCycler™ Thermal Cycler (BIO-RAD). Amplification products were checked through electrophoresis with a 1.5% (*w*/*v*) agarose gel stained with RedSafeTM (Intron Biotechnology) and visualized under ultraviolet light. A 100 bp DNA Ladder GTP (gTPbio) was used as a molecular weight marker. Subsequently, the PCR products were purified using a MEGAquick-spinTM Total Fragment DNA Purification kit (INTRON Biotechnology) following the manufacturer’s instructions. The resulting amplicons were sequenced in both directions by the Central Research Support Service (CRSS; SCAI in Spanish) of the UCO (Spain).

#### 2.4.3. Blast Search

For each isolate and locus (Fungi: ITS, TUB, and LSU; Bacteria: 16S rDNA), the consensus sequence (from sequences’ forward and reverse primer) was obtained in a single FASTA file format, using SeqMan software (DNASTART Lasergen SeqMan^®^ v. 7.0.0, Madison, WI, USA). These consensus sequences were directly BLAST-searched in GenBank (http://www.ncbi.nlm.nih.gov/genbank/ (accessed on 25 March 2020)). Sequences derived in this study were logged with GenBank, and their GenBank accession numbers are shown in Table 2 (ITS and 16S rDNA) or in the Results section (TUB and LSU).

### 2.5. Phenotypic Characterization of Fungal Isolates

#### 2.5.1. Fungal Colonies and Conidia Morphology

Colony morphology was evaluated after growing the 16 fungal isolates on PDA or the yeast isolate (MZC-19) on NA after 7 days of incubation at 25 ± 2 °C in the dark. At that moment, the characteristics of mycelia (texture, density, margin, and zonation) were recorded [35] and colony colour was also determined with a colour scale [36]. For conidial measurements, all fungal isolates were grown on PDA at 25 ± 2 °C for 7 days under continuous fluorescent light (350 μmol m^–2^ s^–1^). Subsequently, conidial masses obtained directly from the fungal colony grown on PDA or NA were placed on slides with a drop of 0.01% acid fuchsine in lactoglycerol (1:2:1 lactic acid:glycerol:water), and covered with a coverslip. Fungal structures were measured at 1000× magnification under oil immersion objective using a Nikon Eclipse 80i microscope (Nikon Corp., Tokyo, Japan). For each isolate, 30 conidia were measured, and the average length and width were calculated. The length/width ratio was also obtained for conidia. 

#### 2.5.2. Effect of Temperature on Mycelial Growth

The 16 fungal isolates identified in this study (excluding the yeast isolate MZC-19) were grown on PDA at 25 °C in the dark for 14 days. Mycelial plugs (7.5 mm diameter) obtained from the margins of actively growing colonies on PDA as described above were placed in the centre of Petri dishes filled with PDA. The Petri dishes were incubated at 5, 10, 15, 20, 25, 30, 35, and 40 °C in the dark. The largest and smallest diameters of the colonies were measured with a digital scale ruler from 4 to 8 days after inoculation, depending on the mycelial growth development of each isolate. Mean data were converted to radial growth rate (mm day^−1^). There were four replicate Petri dishes per isolate and temperature combination, and the experiment was conducted twice. For each isolate, a nonlinear adjustment of the data was applied using the generalized analytics beta model [37] to evaluate the variation in mycelial growth rate with temperature, and the optimum growth temperature and the maximum growth rate (MGR) were obtained as described by López-Moral et al. [38]. Data were tested for normality and homogeneity, and analysis of variance (ANOVA) was performed with the optimum growth temperature or the MGR as the dependent variables and isolate as the independent variable. For each variable, isolate means were compared according to protected Tukey’s HSD test at *p* = 0.05 [39]. Data from this experiment were analysed using Statistix 10 software [40].

### 2.6. Phenotyphic and Biochemical Characterization of Bacterial Isolates

The colony pigmentation of the six bacterial isolates was described on NA after growing at 25 ± 2 °C for 48 h in the dark. At that moment, the bacterial colonies were subjected to Gram staining techniques and observed at 1000× magnification as described above to determine the Gram reaction [41], as well as the cell morphology, endospore formation, and arrangement of the cells. 

### 2.7. Evaluation of Cellulolytic Activity 

The cellulolytic activity of the 17 fungal and the six bacterial isolates identified in this study was evaluated on carboxymethylcellulose agar medium (CMC; carboxymethylcellulose, 10 g; CaCl_2_, 0.1 g; yeast extract, 0.5 g; (NH_4_)_2_SO_4_, 0.5 g; KH_2_PO_4_, 1 g; agar, 12 g, in 800 mL deionized water) [42]. 

For fungal isolates, mycelial plugs (7.5 mm diameter) obtained from the margin of actively growing colonies on AS at 25 ± 2 °C for 7 days in the dark were placed in the centre of Petri dishes with CMC, and they were incubated until fungal hyphae covered 50% of the Petri dish surface [3,43]. For bacterial and yeast isolates, they were previously grown on NA at 25 ± 2 °C for 48 h in the dark, and subsequently, they were inoculated in streak on CMC. *Penicillium chrysogenum* isolate MZC-0 (Table 2) and *Escherichia coli* isolate CECT 102; NCIMB 9483 (from ‘Colección Española de Cultivos Tipo’, ‘Universitat de València’, Valencia, Spain) were included as positive and negative control, respectively, also grown on CMC. These two species were selected as controls based on their cellulolytic activity reported in the literature [44,45]. In all cases, there were three replicated Petri dishes per isolate. All Petri dishes were incubated at 25 ± 2 °C in the dark for 2 to 7 days, depending on the growth rate of each isolate.

After incubation, the colonized surface of the CMC Petri dishes was flooded with Gram’s iodine solution, and the cellulolytic activity of all fungal and bacterial isolates was firstly assessed determining the presence or absence of a hydrolysis halo surrounding the colonies [46,47,48]. Because no halo appeared surrounding the colonies of all the bacterial and yeast isolates growing on CMC, the cellulolytic activity of these isolates could not be estimated. For the 16 fungal isolates plus the control MZC-0, the cellulolytic activity was quantified by means of the enzymatic index (EI) [45,49]. To this end, the largest and smallest diameters of the colony and the hydrolysis halo surrounding the colony (hydrolysis zone) of each isolate were measured with a digital scale ruler, and mean data of each diameter were obtained. The EI was calculated from the ratio between the diameter of the hydrolysis zone and the diameter of the colony. The isolates that showed an EI > 1.0 were considered to be potential producers of cellulases. Data of radial growth rate and EI were subjected to ANOVA to determine differences between fungal isolates for each variable. Additionally, mean data of the diameters of the colonies were converted to radial growth rate (mm day^−1^) to determine this parameter of each isolate in CMC. EI and radial growth rate data were tested for normality and homogeneity of variances, and then they were subjected to ANOVA. In both cases, treated means were compared according to protected Tukey’s HSD test at *p* = 0.05 [39]. Data from this experiment were analysed using Statistix 10 software [40].

## 3. Results

### 3.1. Field Sampling, Insect Collection, and Microorganism Isolation

A total of 23 microorganism isolates were obtained: six out of 23 isolates (26.1%) were obtained from wood degraded by *R. grassei* in the field, three out of 23 (13.0%) from termites’ exoskeleton surface resuspension, and 14 out of 23 from sapwood or filter paper exposed to termites in the laboratory (60.9%).

### 3.2. Microorganism Dispersion by the Termites under Controlled Conditions

*Assay I.* Macroscopic fungal structures were observed only in those cellulose sources that were in contact with termites after one week of incubation. However, both fungi and bacteria were isolated from the different food sources exposed to the termites, with fungi (64.3%) being isolated more frequently than bacteria (35.7%) (see Table 2; microorganisms recovered from the source referred as ‘Filter paper or Wood/Laboratory colonization’). No microorganisms were isolated from the sterilized substrates not exposed to termites (control). 

**Figure 1:**
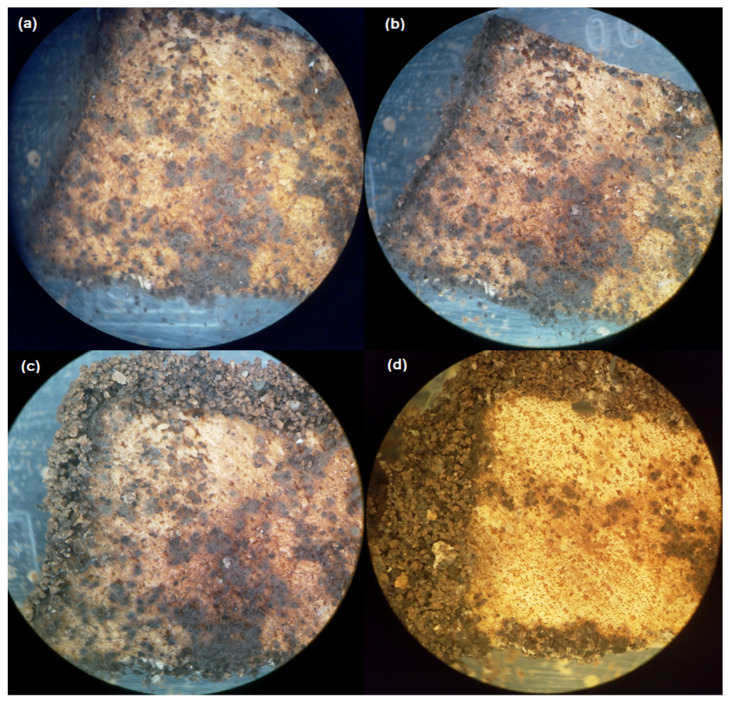
Progressive degradation of fungal colonies by *Reticulitermes grassei* on colonized wood substrates under laboratory conditions. (**a**) At the beginning of the experiment, and at (**b**) 24 h, (**c**) 48 h, and (**d**) 120 h after wood exposure to *R. grassei* in hermetic plastic containers. Please note the margins of the wood block covered with vermiculite by the activity of termites at 48 and 120 h after wood exposure to *R. grassei*.

*Assay II.* The interaction between *R. grassei* and the fungi on the food sources previously exposed to the termites resulted in a progressive degradation of the fungal mycelial that covered the cellulose sources (Figure 1).

### 3.3. Molecular Characterization

Fungal isolates: based on ITS sequences, the following fungal species were identified: *Aspergillus flavus* (isolates MCZ-17 and MCZ-20), *A. niger* (isolates MCZ-14 and MCZ-18), *Chaetomium globosum* (isolates MCZ-2 and MCZ-9), *Doratomyces stemonitis* (isolate MCZ-16), *Mucor circinelloides* (isolates MCZ-5 and MCZ-23), *M. fragilis* (isolate MCZ-1), *Penicillium citrinum* (isolate MCZ-21), *Pichia guilliermondii* (yeast; isolate MCZ-19), *Trichoderma asperellum* (isolates MCZ-8, MCZ-10, MCZ-11, and MCZ-24), and *T. longibrachiatum* (isolate MCZ-4) (Table 2). Based on the blast of TUB reference sequences, the identity of the isolates MZC-17 (GenBank Accession Number: MN095349) and MZC-20 (Acc. Nº: MN095350) was confirmed as *A. flavus* with the reference sequence of *A. flavus* CICC 40,186 (Acc. Nº: KX462752; identity = 99.8%); the isolates MZC-14 (Acc. Nº: MN095351) and MZC-18 (Acc. Nº: MN069571) as *A. niger* with the reference sequence of *A. niger* 1062 (Acc. Nº: KM189806; identity = 100%); and the isolate MZC-21 (Acc. Nº: MN095352) as *P. citrinum* with the reference sequence of *P. citrinum* a2s6_6 (Acc. Nº: KC345003; identity = 100%). Finally, the identity of the isolates MZC-2 (Acc. Nº: MN099959) and MZC-9 (Acc. Nº: MN069565) was confirmed as *C. globosum* with the LSU reference sequence of *C. globosum* MUT<ITA>:4942 (Acc. Nº: KP671732; identity = 99.6%).

Bacterial isolates: based on the blast of the 16S rDNA sequences of our bacterial isolates, the following species were identified: *Burkholderia kirstenboschensis* (isolate MCZ-3), *Klebsiella aerogenes* (isolate MCZ-13), *Lactococcus lactis* subsp. *cremoris* (isolate MCZ-7), and *Serratia marcescens* (isolates MCZ-6, MCZ-12, and MCZ-22) (Table 2).

### 3.4. Phenotypic Characterization of Fungal Isolates

#### 3.4.1. Fungal Colonies and Conidia Morphology

**Figure 2:**
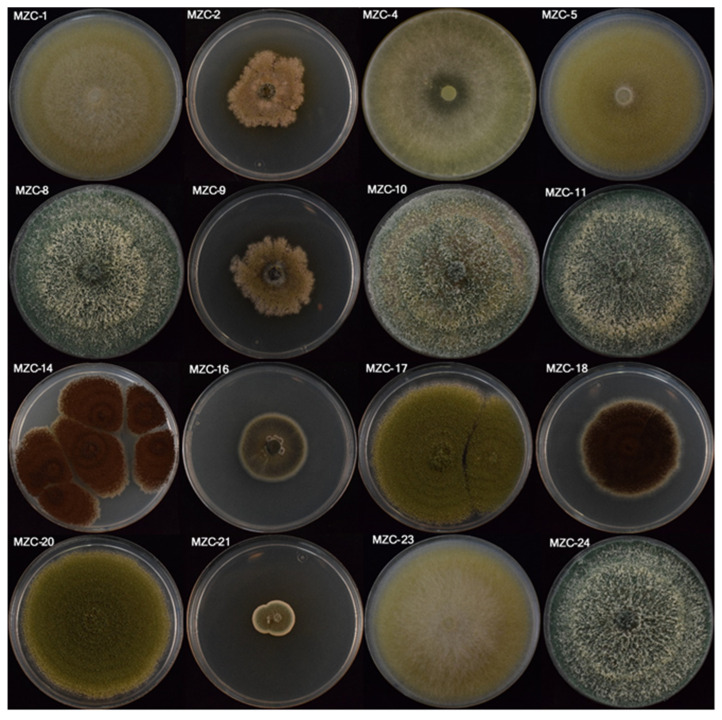
One-week-old colonies growing on PDA at 25 ± 2 °C in the dark of the 16 fungal isolates obtained from degraded wood by the subterranean termites *Reticulitermes grassei* (For interpretation of the references of the isolates, the reader is referred to Table 2).

Most of the fungal isolates showed floccose to felted fast-growing aerial mycelium varying in colour on PDA, with regular margin and without zonation (Table 3; Figure 2). In general, the 16 fungal isolates evaluated showed hyaline, aseptate, abundant, small, rounded to ovoid conidia (Table 4). It should be noted that the yeast isolate MZC-19 differed markedly in appearance regarding the remaining fungal isolates because it did not develop filamentous mycelia on PDA. Thus, it was transferred to NA due to its bacteria-like appearance. In this medium, the isolate MZC-19 showed a moist, smooth, and cream to yellow colony (Table 3); and aseptate, abundant, small, rounded to ovoid, hyaline cells (Table 4). 

#### 3.4.2. Effect of Temperature on Mycelial Growth

A total of 16 fungal isolates identified in this study (excluding the yeast isolate MZC-19) were used in this experiment. All of them were able to grow on PDA from 10 to 30 °C, showing little or sometimes lacking mycelial growth development at the minimum or maximum temperatures tested of 5 and 35 °C, respectively. Significant differences in optimum growth temperature and MGR (*p* < 0.0001 in all cases) were observed between species, and eventually, between isolates within a genus or between isolates of the same species, mainly for optimum growth temperature. Optimum growth temperatures ranged from 22.2 to 32.1 °C for *C. globosum* isolate MZC-9 and *T. longibrachiatum* isolate MZC-4, respectively. MGR ranged from 1.8 to 33.6 mm day^−1^, with *P. citrinum* isolate MZC-21 having the lowest MGR and *T. longibrachiatum* isolate MZC-4 the highest MGR (Table 5).

### 3.5. Phenotyphic and Biochemical Characterization of Bacterial Isolates

**Figure 3:**
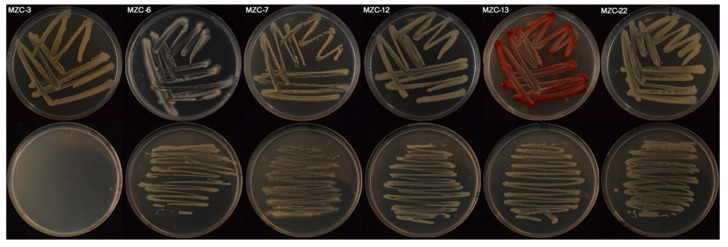
Two-day-old colonies growing on PDA (top) and NA (bottom) at 25 ± 2 °C in the dark for 7 days of the six bacterial isolates characterized in this study and collected from degraded wood by the subterranean termites *Reticulitermes grassei* (For interpretation of the references of the isolates, the reader is referred to Table 2).

The colonies of the six bacterial isolates were large mucoides, and their pigmentation depended on the composition of the culture medium used [50]. In NA, *Burkholderia* isolate MZC-3 was colourless, whereas the remaining isolates showed creamy yellow colonies; in PDA, *Klebsiella* isolate MZC-13 showed pink colonies, and the remaining isolates showed creamy yellow colonies (Figure 3). Five of the six bacterial isolates evaluated were Gram-negative, rod-shaped, single, in pairs or short chains, and non-spore-forming bacteria (*Burkholderia kirstenboschensis* isolate MZC-3, *Klebsiella aerogenes* isolate MZC-13, and *Serratia marcescens* isolates MZC-6, MZC-12, and MZC-22). *Lactococcus lactis* subsp *cremoris* isolate MZC-7 was Gram-positive cocci nonsporulated, in pairs and short chains. 

### 3.6. Evaluation of Cellulolytic Activity

**Figure 4:**
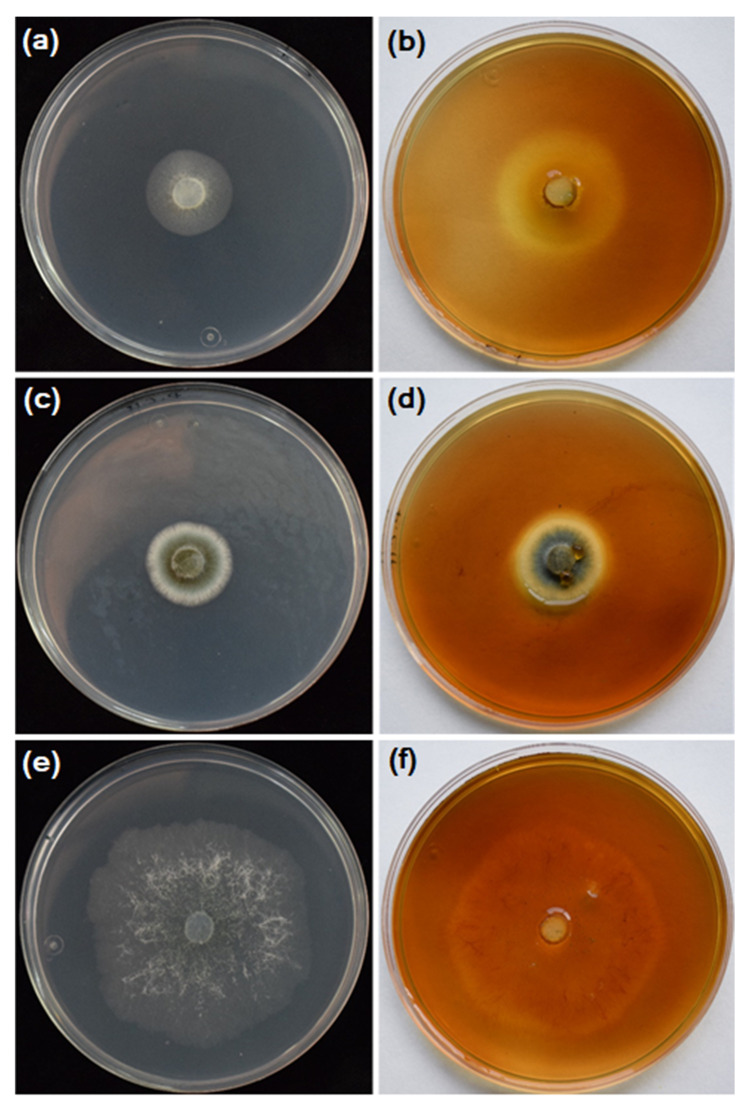
Cellulose degradation ability shown by several fungal isolates obtained from degraded wood by the subterranean termites *Reticulitermes grassei*. Fungal colonies of (**a**,**b**) *Penicillium chrysogenum* isolate MZC-0 (positive control), (**c**,**d**) *P. citrinum* isolate MZC-21, and (**e**,**f**) *Trichoderma longibrachiatum* grown on carboxymethylcellulose at 25 ± 2 °C in the dark for 96 and 36 h for *Penicillium* isolates and *T. longibrachiatum*, respectively; (**a**,**c**,**e**) before and (**b**,**d**,**f**) at 5 min after flooding with Gram’s iodine solution.

There were significant differences for both ‘radial growth rate’ and EI dependent variables between fungal species and between isolates (*p* < 0.0001 in all cases). In general, all the studied isolates showed average to fast mycelial radial growth ranging from 10.9 ± 0.15 to 1.6 ± 0.17 mm day^−1^ for *T. longibrachiatum* isolate MZC-4 and *P. citrinum* isolate MZC-21, respectively. Concerning the EI, the positive control *P. chrysogenum* isolate MZC-0 (+) showed the highest EI value (1.50 ± 0.011), followed by *P. citrinum* isolate MZC-21 (EI = 1.34 ± 0.020). The isolates *A. niger* MZC-18, *C. globosum* MZC-2 and MZC-9, *M. circinelloides* MZC-5 and MZC-23, *M. fragilis* MZC-1, and *T. asperellum* MZC-8, MZC-10, MZC-11, and MZC-24 showed an EI = 1.0. The remaining isolates showed EI values above 1.0, which ranged from 1.05 to 1.09 for *D. stemonitis* isolate MZC-16 and *T. longibrachiatum* isolate MZC-4, respectively. Despite the fact that several fungal isolates did not develop a halo surrounding the colony, all of them were considered to be cellulase producers since they were able to grow on CMC (Table 6; Figure 4).

## 4. Discussion

Field sampling was conducted in order to collect microorganisms associated with the subterranean termite *R. grassei* in natural colonized ecosystems in the Andalusia region (southern Spain). The 23 microorganism isolates were characterized morphologically and molecularly before testing their cellulolytic activity. Morphological characteristics were useful to identify most of the isolates at the genus level according to the literature. However, molecular tools were needed to confirm the identity of the 23 isolates at the species level. The following species were identified: fungi: *Aspergillus flavus*, *A. niger*, *Chaeotomium globosum*, *Doratomyces stemonitis*, *Mucor circinelloides*, *M. fragilis*, *Penicillium citrinum*, *Pichia guilliermondii* (yeast isolate MCZ-19), *Trichoderma asperellum*, and *T. longibrachiatum*; bacteria: *Burkholderia kirstenboschensis*, *Klebsiella aerogenes*, *Lactococcus lactis* subsp. *cremoris*, and *Serratia marcescens*.

In general, fungi were the most prevalent microorganisms (72.7%) associated with *R. grassei*, with bacteria showing a minor relevance (27.3%). This could be because bacteria associated with the activity of termites have been traditionally isolated from the gut of termites such as *Microcerotermes diversus*, *R. grassei,* or *R. lucifugus*, among other invertebrates including snails, caterpillars, and bookworms [51,52,53,54], and most of our isolates were collected from cellulose substrate, i.e., wood attacked by *R. grassei*, cellulose food sources offered in the laboratory, etc. Therefore, we focused our study on identifying the interaction between fungi and *R. grassei*.

The effect of temperature on mycelial growth showed optimum growth temperatures ranging around 27 and 31 °C for most of the fungal isolates. Note that this characteristic suggests that those fungal isolates associated with *R. grassei* could be well adapted to survive and develop in warm regions such as southern Spain. The climate of this geographic area is typically Mediterranean, with winter rainfall between 500 and 800 mm per year, average annual temperature around 17 °C, and relatively warm summers with average temperature around 24 °C [22].

Our results for the cellulolytic activity observed in all the fungal isolates show similarities with those obtained by several authors on other termites worldwide. The genera *Aspergillus*, *Mucor*, *Penicillium*, and *Trichoderma* have already been isolated from other species of termites around the world such as *Globitermes sulphureus* in Malaysia [55], *Nasutitermes corniger* in Brazil [56], and *Reticulitermes* spp. in North America [57,58]. Our study represents the first report of *D. stemonitis* isolated from termites. This species is considered to be a saprotrophic fungus belonging to the mycobiota of the cereal rhizosphere [59]. 

The presence of these fungi in the natural environment of *R. grassei* could bring it nutritional benefits, defence against termite parasites, and advantages over others wood -organisms [20]. For example, it increases the availability of nutrients, detoxifies wood, and improves the ability of termites to metabolize cellulose through physical and chemical modification of the wood [60]. Nevertheless, depending on the fungal species and the parameters used to evaluated the relationship between fungi and termites, this effect can be null, but not negative, suggesting that there are more mutualistic interactions than antagonistic ones [61].

Fungal species belonging to the *Trichoderma* genus show different responses depending on if they interact with higher or lower termites. In higher termites, *Trichoderma* spp. act by repelling fungus-growing termites such as *Odontotermes formosanus* Shiraki, because they develop an antagonistic interaction with *Termitomyces* fungi, cultivated by *O. formosanus* to produce its own food. However, *Trichoderma* fungi act by attracting lower termites such as *Coptotermes formosanus* [62] because they can inhibit the growth of wood-rotting fungi such as *Gloeophyllum trabeum*, which competes with termites for cellulose sources [63]. To sum up, the attractive effect of the *Trichoderma* genus against lower termites could be related to their ability to produce antifungal compounds [64,65,66] as well as their ability to degrade cellulose [67,68]. Similar processes could occur in the interaction between *C. globosum* and *R. grassei*, since several authors have also described the capacity of this fungus to produce antifungal compounds [69,70] and cellulases [71,72].

Concerning the association between *Aspergillus* spp. and *R. grassei*, this could be related to their cellulolytic ability and their capacity for inhibiting wood-rotting fungi [63]. *Aspergillus flavus* is considered entomopathogenic of a broad diversity of insect species, including honeybees, in which it causes a disease called stonebrood [73,74]. However, host specialization has never been observed for *A. flavus* strains [75]. In addition to the entomopathogenic ability of *A. flavus* and *A. niger*, these fungi are aflatoxin (a potent carcinogen) producers [76,77]. However, further studies on the interaction between these fungi and *R. grassei* are needed to elucidate the effect of *Aspergillus* spp. aflatoxins in lower termites.

Some of the fungal species described in this study have been characterized by their ability to accumulate fatty acids, such as *M. circinelloides* [78,79], *M. fragilis* [80], and *P. citrinum* [81]. In this case, the lower termites could establish synergistic interactions with these fungi for the benefits of lipid intake to complement their poor diet. Concerning *D. stemonitis*, it is interesting to note that this species was described by Peterson et al. [82] as a coprophilous fungus inhabiting in herbivore faeces. This fungus can secrete enzymes that are able to degrade the most recalcitrant parts of plant biomass that resist the herbivore digestive process. However, it is still uncertain if the association of this fungus with *R. grassei* is related to its own faeces or the wood that the termite eats. Regarding *P. citrinum*, it is described as a mycotoxin producer, with citrinin being the most common. Li et al. [83] analysed the citrinin production of this fungus with different carbon sources, with the results showing a major production in the presence of glucose rather than sucrose. This fact, linked with our results and those of Bardhan et al. [81], who tested the cellulolytic ability of some strains of *P. citrinum* and their lipid production, supports our hypothesis of termite feeding behaviour on fungi for nutritional requirements. However, the potential effect of citrinin as attractive or repellent to termites is unknown.

The benefits that cellulolytic fungi provide to *R. grassei* seem clear, but how do termites benefit fungi? The following facts shed some light. (i) Some of the fungi described here have already been isolated from the gut of closely related species to *R. grassei* such as *R. flavipes* [58]; (ii) our observations from the dispersion assay conclude that termites acted by inducing the formation of vegetative and reproductive fungal structures on food substrates, which were subsequently eaten by *R. grassei*; (iii) termite nests seem to be ideal places for fungal growth due to their pH [84] and nutritional conditions [85]; and (iv) it has been demonstrated that *Reticulitermes* species contribute significantly to the dispersion of fungi such as *Beauveria bassiana* [86].

All these facts lead us to reinforce our hypothesis on that the microorganisms described here are dispersed by *R. grassei*. However, further studies are needed to understand this behaviour, and to clarify if the dispersion is achieved by means of endozoochory, exozoochory, or both simultaneously. Moreover, all the data described above could also explain our observations in *Assay II*, in which fungi developed on previously colonized food sources were degraded progressively when they were exposed to *R. grassei*. However, further research is needed to demonstrate if this is caused by feeding or by hygiene behaviour. 

The 16 fungal isolates identified in this study were able to grow on CMC agar as the only carbon source, so it can be concluded that all they are cellulase producers. Among them, *Trichoderma* isolates showed the highest values of MGR on CMC, with *T. longibrachiatum* (MZC-4) showing the highest values. Our results are in concordance with those obtained by other authors who compare these species with others used in the bioindustry [62,67] or who characterized cellulolytic microorganisms isolated from an art collection [45]. Species belonging to the *Mucor* genus (MZC-1, MZC-5, and MZC-23) also showed high values of MGR on CMC. 

The EI values from our isolates were similar to those obtained by Coronado-Ruiz et al. [45], except for that showed by *P. chrysogenum*, from which these authors obtained a higher EI (3.3) in comparison with our results (EI = 1.5). Although the EI values obtained from our isolates were slightly lower than those observed by other authors, we must consider that the method used here is useful to determine the capacity of the microorganisms to produce cellulase, but not to quantify it. In fact, Coronado-Ruiz et al. [45] indicate that just the ability of the microorganisms to show a high radial growth rate on CMC is enough to conclude that they are cellulase producers. Likewise, all our fungal isolates showed major radial growth on CMC, significantly higher than that observed for *P. chrysogenum* isolate MCZ-0 (positive control), except for *P. citrinum* isolate MZC-21. It is interesting to note that these two isolates showed the least radial growth in the whole of the experiment, but the highest halo. This could be due to the fact that fungal species with lower radial growth need to develop mechanisms to degrade cellulose to supply their minor capacity to colonize living tissues. Florencio et al. [49] suggest that the diameter of the hydrolysis zone is useful for selecting isolates that can efficiently degrade polysaccharides such as cellulose. In addition, there is a broad diversity of studies in the literature that conclude that determining the radial growth and EI parameters on CMC or even growth with this compound as the sole carbon source is enough to have a first approach about the cellulolytic activity of microorganisms [54,87,88]. However, further studies will be conducted in the future to evaluate specific biochemical tests such as some lignin- and xylan-degrading enzyme assays to determine the highest cellulase producers by quantitative methods [89,90].

The six bacterial isolates and the yeast isolate MCZ-19 were able to grow on CMC; however, since no halo appeared surrounding their colonies, further and more specific studies for these isolates should be carried out to measure their cellulolytic activity in the future.

It seems that cellulolytic fungi have a symbiotic relationship with *R. grassei* since the termites make their dissemination to new habitats with optimal conditions easier, such as termite nests and the foraging zones. The habitats associated with the activity of the subterranean termites are characterized by high levels of humidity and moderate temperatures, with plenty of cellulose and slightly acid substrate availability [84]. These conditions are highly favourable for fungal growth and the reproduction of some species [91]. In parallel, the presence of cellulolytic fungi in the termites ecosystems allow nutritional (supplementing their poor diet) and hygienical (preventing the proliferation of opportunistic, parasitic fungi) benefits for the subterranean termites. Likewise, termites and their immediate environment (i.e., nests, degraded substrates, etc.) are a potential target to collect new cellulolytic microorganism isolates, with biotechnological potential in fields such as biofuel production and the food industry. Altogether, this work is relevant since it contributes to generating knowledge and creativity based on the potential benefits from the natural association between *R. grassei* and cellulolytic fungi, and their interaction. However, further research is needed to go on to elucidate both negative and positive effects of the interaction between *R. grassei* and cellulolytic fungi in Mediterranean ecosystems, and their properties.

## Figures and Tables

**Table 1:** Primer pairs and PCR conditions used for the amplification of the genes included in this study.

Gene ^1^	Primer Pairs ^2^	PCR Cycling Program (T-Time)					
		Initial Denaturation	Amplification				Final Extension
			N° of Cycles	Denaturation	Annealing	Extension	
Fungal isolates							
ITS	ITS4/ITS5	94 °C-3 min	35	94 °C-30 s	48 °C-30 s	72 °C-45 s	72 °C-10 min
TUB	Bt2a/Bt2b	95 °C-3 min	35	94 °C-15 s	55 °C-15 s	72 °C-1 min	72 °C-7 min
LSU	LR0R/LR7	95 °C-3 min	35	94 °C-15 s	52 °C-15 s	72 °C-1 min	72 °C-7 min
Bacterial isolates							
16S rDNA	27F/1492R	94 °C-3 min	30	94 °C-30 s	55 °C-30 s	72 °C-1 min	72 °C-10 min

^1,2^ ITS = internal transcribed spacer 1–5.8S rDNA-internal transcribed spacer 2 [30]; TUB = partial β-tubuline gen [31]; LSU = partial 28S rDNA gene [32,33]; 16S rDNA gene [34].

**Table 2:** List of microorganisms associated with the activity of the subterranean termite *Reticulitermes grassei*, with their corresponding GenBank accession numbers and data of Blast results obtained from GenBank.

Species	Isolate ^1^	Host (Plant)	Substrate/Conditions ^2^	Genbank Accession ^3^	Blast Accession ^4^	Query Length	Gaps ^5^	Identities ^6^	Maximum Identity (%)
Internal Transcribed Spacer Region Gene (Fungal and Yeast Isolates)									
*Aspergillus flavus*	MZC-17	*Eucalyptus* sp.	Wood/Field colonization	MN069570	KX462773	589	1/584	58/3/584	99.8
	MZC-20	*Pinus pinea*	Wood/Laboratory colonization	MN069573	MH485381	586	0/581	581/581	100
*Aspergillus niger*	MZC-14	*Pinus pinea*	Wood/Laboratory colonization	MN069568	KM259827	626	1/599	599/599	100
	MZC-18	*Pinus pinea*	Wood/Laboratory colonization	MN069571	KM259827	623	0/623	623/623	100
*Chaetomium globosum*	MZC-2	-	Filter paper/Laboratory colonization	MN069561	KR014363	603	1/496	493/493	100
	MZC-9	-	Filter paper/Laboratory colonization	MN069565	KM268665	606	0/602	602/602	100
*Doratomyces stemonitis*	MZC-16	*Populus alba*	Wood/Laboratory colonization	MN069569	FR799473	588	2/588	583/588	99.1
*Mucor circinelloides*	MZC-5	*-*	Suspension from collected termites	MN069563	HM641688	668	1/657	650/657	99.0
	MZC-23	*Populus alba*	Wood/Laboratory colonization	MN069575	JN561250	627	1/627	624/627	99.5
*Mucor fragilis*	MZC-1	*-*	Suspension from collected termites	MN069560	GU566275	664	1/662	659/662	99.5
*Penicillium chrysogenum*	MZC-0 ^7^	*-*	-	MN069559	KC009774	612	2/612	610/612	99.7
*Penicillium citrinum*	MZC-21	*Pinus pinea*	Wood/Laboratory colonization	MN069574	KC344966	538	0/538	538/538	100
*Pichia guilliermondii*	MZC-19 (Yeast)	*Pinus pinea*	Wood/Laboratory colonization	MN069572	EF192233	631	4/621	616/621	99.1
*Trichoderma asperellum*	MZC-8	*Eucalyptus* sp.	Wood/Field colonization	MN069564	KM268676	622	0/615	615/615	100
	MZC-10	*Eucalyptus* sp.	Wood/Field colonization	MN069566	EU280110	631	1/629	627/629	100
	MZC-11	*Eucalyptu*s sp.	Wood/Field colonization	MN069567	MH351209	591	0/589	588/589	99.8
	MZC-24	*Eucalyptus* sp.	Wood/Field colonization	MN069576	EU280110	631	2/621	618/621	99.5
*Trichoderma longibrachiatum*	MZC-4	*Eucalyptus* sp.	Wood/Field colonization	MN069562	KJ174230	622	2/623	621/623	99.7
16S rDNA gene (Bacteria isolates)									
*Burkholderia kirstenboschensis*	MZC-3	*Populus alba*	Wood/Laboratory colonization	NM069326	HF674708	1367	2/1366	1353/1366	99.0
*Klebsiella aerogenes*	MZC-13	*-*	Suspension from collected termites	NM069330	MH368390	1391	1/1391	1389/1391	99.9
*Lactococcus lactis* subsp. *cremoris*	MZC-7	-	Filter paper/Laboratory colonization	NM069328	MK290341	1006	0/1006	1005/1006	99.9
*Serratia marcescens*	MZC-6	*Pinus pinea*	Wood/Laboratory colonization	NM069327	CP018929	1397	0/1397	1397/1397	100
	MZC-12	*Pinus pinea*	Wood/Laboratory colonization	NM069329	MK157269	1012	1/1012	1008/1012	99.6
	MZC-22	*Pinus pinea*	Wood/Laboratory colonization	NM069331	KR817904	1403	1/1403	1401/1403	99.9

^1^ MZC: Collection of Microbiology and Zoology Departments of UCO of cellulolytic microorganisms. ^7^ Positive control in cellulolytic activity test. ^2^ Substrates and conditions from which microorganisms associated with *Reticulitermes grassei* were isolated: from decomposed wood of forest trees colonized by *R. grassei* (Wood/Field colonization); from food sources after being eaten by *R. grassei* in laboratory-controlled conditions (Filter paper or Wood/Laboratory colonization), or from a suspension of collected termites (*R. grassei*) from affected fields (Suspension from collected termites). ^3^ Corresponding GenBank accession numbers of our isolates. ^4^ GenBank accession numbers blasted with the isolates obtained in this study. ^5^ Number of spaces introduced into the alignment to compensate for insertions and deletions in our sequence relative to blasted sequences. ^6^ Number of nucleotides of our sequences/number of nucleotides of blasted sequences.

**Table 3:** Phenotypical characteristics of the colonies of the fungal isolates included in this study.

Isolate ^1^	Mycelium				
	Obverse			Reverse	
	Colour	Zonation	Margin	Colour	Zonation
MZC-1	Beige to light brown	No	Regular	Beige	No
MZC-2	Light grey, white in the margins	No	Irregular	Dark grey, beige in the margin	No
MZC-4	White, yellow pigmentation of culture medium	No	Regular	Yellow	No
MZC-5	Beige to light brown	No	Regular	Beige	No
MZC-8	White to dark green	No	Irregular	White to green-grey	No
MZC-9	Light grey, white in the margins	No	Irregular	Dark grey, beige in the margin	No
MZC-10	White to dark green	No	Irregular	White to green-grey	No
MZC-11	White to dark green	No	Irregular	White to green-grey	No
MZC-14	Black, white in the margins	No	Regular	White to light grey	No
MZC-16	Olive green, white in the margins	No	Regular	Olive green, white in the margins	No
MZC-17	Yellow-green, white in the margins	No	Regular	Beige to light brown	No
MZC-18	Black, white in the margins	No	Regular	White to light grey	No
MZC-19 ^2^	Cream to yellow	-	-	Cream to yellow	-
MZC-20	Yellow-green, white in the margins	No	Regular	Beige to light brown	No
MZC-21	Green-grey, white in the margins	No	Regular	Yellow-white	No
MZC-23	Beige to light brown	No	Regular	Beige	No
MZC-24	White to dark green	No	Irregular	White to green-grey	No

^1^ Single conidial cultures were grown on PDA for 7 days at 25 ± 2 °C in the dark, and colony colour was determined by means of a colour chart [36]. ^2^ Yeast isolate grown on NA for 7 days at 25 ± 2 °C under continuous fluorescent light (350 μmol m^–2^ s^–1^).

**Table 4:** Morphological characteristics of conidia of the fungal isolates included in this study.

Isolate	Conidia			
	Length × Width (µm) ^1^	Length/Width ^2^	Morphology	Colour
MZC-1	(3.9-) 6.1 (−8.8) × (2.6-) 4.2 (−6.7)	1.51 ± 0.05	Aseptate, abundant, small, rounded to ovoid	Hyaline
MZC-2	(7.0-) 8.6 (−10.1) × (5.6-) 7.2 (−8.8)	1.20 ± 0.02	Aseptate, small, rounded to ovoid, thick wall, pointed ends	Hyaline
MZC-4	(2.6-) 3.6 (−5.9) × (2.1-) 2.6 (−3.1)	1.38 ± 0.06	Aseptate, abundant, small, rounded to ovoid	Hyaline
MZC-5	(4.3-) 5.4 (−6.5) × (4.0-) 4.9 (−5.6)	1.13 ± 0.03	Aseptate, abundant, small, rounded to ovoid	Hyaline
MZC-8	(2.7-) 3.5 (−4.2) × (2.4-) 3.0 (−3.5)	1.17 ± 0.04	Aseptate, abundant, small, rounded to ovoid	Hyaline
MZC-9	(7.1-) 8.7 (−10.3) × (4.5-) 6.7 (−8.2)	1.32 ± 0.03	Aseptate, small, rounded to ovoid, thick wall, pointed ends	Hyaline
MZC-10	(2.7-) 3.3 (−4.2) × (2.2-) 2.6 (−3.7)	1.17 ± 0.03	Aseptate, abundant, small, rounded to ovoid	Hyaline
MZC-11	(2.5-) 3.3 (−4.1) × (2.2-) 2.8 (−3.4)	1.20 ± 0.03	Aseptate, abundant, small, rounded to ovoid	Hyaline
MZC-14	(2.8-) 3.9 (−4.9) × (3.1-) 3.8 (−5.4)	1.05 ± 0.03	Aseptate, abundant, small, rounded to ovoid	Hyaline, dark grey cell centre
MZC-16	(4.3-) 5.3 (−6.7) × (2.7-) 3.7 (−4.6)	1.47 ± 0.03	Aseptate, small, ovoid, thick double wall, one rounded end, and other truncate	Hyaline
MZC-17	(2.7-) 3.6 (−5.3) × (2.4-) 3.3 (−4.0)	1.09 ± 0.02	Aseptate, abundant, small, rounded to ovoid	Hyaline
MZC-18	(3.3-) 3.7 (−4.2) × (3.3-) 3.7 (−4.3)	1.00 ± 0.02	Aseptate, abundant, small, rounded to ovoid	Hyaline, dark grey cell centre
MZC-19 ^3^	(2.4-) 4.0 (−8.2) × (1.3-) 2.0 (−2.5)	2.11 ± 0.14	Aseptate, abundant, small, rounded to ovoid	Hyaline
MZC-20	(2.6-) 3.4 (−4.9) × (2.8-) 3.4 (−4.5)	1.00 ± 0.02	Aseptate, abundant, small, rounded to ovoid	Hyaline
MZC-21	(1.8-) 2.2 (−2.5) × (1.9-) 2.2 (−2.5)	1.00 ± 0.02	Aseptate, abundant, small, rounded	Hyaline
MZC-23	(3.6-) 4.6 (−6.7) × (2.5-) 3.9 (−5.7)	1.22 ± 0.04	Aseptate, abundant, small, rounded to ovoid	Hyaline
MZC-24	(2.8-) 3.4 (−4.3) × (2.5-) 3.1 (−3.8)	1.08 ± 0.03	Aseptate, abundant, small, rounded	Hyaline

^1^ Mean and range values: length × width (µm). The extremes of the conidial measurements are shown inside parenthesis. Values represent the mean of 30 conidia. ^2^ Conidia length/width ratio. Values represent the mean of 30 conidia ± error standard of the mean. ^3^ Yeast isolate grown on NA for 7 days at 25 ± 2 °C under continuous fluorescent light (350 μmol m^–2^ s^–1^).

**Table 5:** Effect of temperature on mycelial growth of the fungal isolates included in this study^1^.

	Isolate ^1^	Analytics Beta Model ^2^			Temperature (°C) ^3,4^			MGR (mm Day^−1^) ^4,5^
		*R* ^2^	a	b	Optimum	Minimum	Maximum	
*Aspergillus flavus*	MZC-17	0.9939	2.27	0.80	31.6 ab	5.0	41.0	5.6 gh
	MZC-20	0.9989	1.79	1.18	30.9 bc	13.5	42.5	5.3 h
*Aspergillus niger*	MZC-14	0.9938	2.63	1.67	31.2 b	10.0	42.5	6.9 g
	MZC-18	0.9845	2.36	1.50	30.3 c	12.0	42.0	6.6 gh
*Chaeotomium globosum*	MZC-2	0.9865	1.74	0.99	24.4 f	5.0	35.5	2.2 i
	MZC-9	0.9668	1.16	0.90	22.2 g	5.0	35.5	2.0 i
*Doratomyces stemonitis*	MZC-16	0.9734	2.10	0.97	24.1 f	6.0	32.5	2.0 i
*Mucor circinelloides*	MZC-5	0.9475	1.97	0.58	25.2 d	3.5	35.5	10.4 e
	MZC-23	0.9621	1.03	0.32	28.3 d	5.0	35.5	11.0 e
*Mucor fragilis*	MZC-1	0.9811	1.47	0.73	24.7 f	4.0	35.0	8.6 f
*Penicillium citrinum*	MZC-21	0.9992	1.24	0.62	27.7 de	12.0	35.5	1.8 i
*Trichoderma asperellum*	MZC-8	0.9970	2.39	0.91	27.8 de	7.5	35.5	13.3 d
	MZC-10	0.9920	3.38	1.18	27.6 de	5.0	35.5	13.9 cd
	MZC-11	0.9915	2.60	0.92	27.5 e	5.0	35.5	15.2 bc
	MZC-24	0.9966	3.58	1.29	27.1 e	5.0	35.5	16.3 b
*Trichoderma longibrachiatum*	MZC-4	0.9957	2.97	0.96	32.1 a	6.0	40.5	33.6 a

^1^ Isolates were grown on PDA at 5, 10, 15, 20, 25, 30, 35, and 40 °C in the dark from 1 to 8 days. Data represent the average of four replicated Petri dishes per isolate and temperature combination. ^2^ Analytics beta model, where *R*^2^ = coefficient of determination and a, b = coefficients of regression. ^3^ For each isolate, temperature average growth rates were adjusted to a regression curve to estimate the optimum growth temperature. ^4^ MGR: Maximum growth rate (mm per day) obtained using the analytics beta model at the optimum growth temperature. ^5^ Means in a column followed by the same letter do not differ significantly according to protected Tukey’s HSD test at *p* = 0.05 [39].

**Table 6:** Cellulolytic activity of the 16 fungal isolates identified in this study associated with the subterranean termite *Reticulitermes grassei*.

Fungal Species	Isolate ^1^	Radial Growth (mm day^−1^) ^2^	Enzymatic Index ^3^
*Aspergillus flavus*	MZC-17	6.6 ± 0.58 e	1.08 ± 0.041 cd
	MZC-20	4.5 ± 0.01 f	1.11 ± 0.019 c
*Aspergillus niger*	MZC-14	5.1 ± 0.25 f	1.06 ± 0.006 cd
	MZC-18	5.0 ± 0.12 f	1.00 ± 0.001 e
*Chaetomium globosum*	MZC-2	3.3 ± 0.17 g	1.00 ± 0.001 e
	MZC-9	3.6 ± 0.11 g	1.00 ± 0.001 e
*Doratomyces stemonitis*	MZC-16	2.3 ± 0.08 h	1.05 ± 0.052 d
*Mucor circinelloides*	MZC-5	7.3 ± 0.87 de	1.00 ± 0.001 e
	MZC-23	9.0 ± 0.36 b	1.00 ± 0.001 e
*Mucor fragilis*	MZC-1	7.3 ± 0.22 de	1.00 ± 0.001 e
*Penicillium chrysogenum*	MCZ-0 ^4^	1.7 ± 0.02 h	1.50 ± 0.011 a
*Penicillium citrinum*	MZC-21	1.6 ± 0.17 h	1.34 ± 0.020 b
*Trichoderma asperellum*	MZC-8	7.5 ± 0.31 cd	1.0 ± 0.001 e
	MZC-10	8.3 ± 0.07 bc	1.0 ± 0.001 e
	MZC-11	8.5 ± 0.22 b	1.0 ± 0.001 e
	MZC-24	7.5 ± 0.15 cd	1.0 ± 0.001 e
*Trichoderma longibrachiatum*	MZC-4	10.9 ± 0.15 a	1.09 ± 0.008 cd

^1^ MZC: Collection of Microbiology and Zoology Departments of UCO of cellulolytic microorganisms. ^2^ Data represent the average of two independent sets (experiments) of three replicated Petri dishes per isolate, each one ± standard error of the means. Means in a column followed by the same letter do not differ significantly according to protected Tukey’s HSD test at *p* = 0.05 [39]. ^3^ Enzymatic index (EI) was calculated as the ratio between the diameter of the hydrolysis halo surrounding the colony and the diameter of the colony. The isolates that showed an *EI* > 1.0 were considered to be potential producers of cellulases. ^4^ Positive control.

## Data Availability

The data that support the findings of this study are available from the corresponding author upon reasonable request.
